# Global holiday datasets for understanding seasonal human mobility and population dynamics

**DOI:** 10.1038/s41597-022-01120-z

**Published:** 2022-01-20

**Authors:** Shengjie Lai, Alessandro Sorichetta, Jessica Steele, Corrine W. Ruktanonchai, Alexander D. Cunningham, Grant Rogers, Patrycja Koper, Dorothea Woods, Maksym Bondarenko, Nick W. Ruktanonchai, Weifeng Shi, Andrew J. Tatem

**Affiliations:** 1 WorldPop, School of Geography and Environmental Science, University of Southampton, Southampton, SO17 1BJ UK; 2 Population Health Sciences, Virginia Tech, Blacksburg, VA 24061 USA; 3 School of Public Health, Shandong First Medical University & Shandong Academy of Medical Sciences, Taian, 271000 China

**Keywords:** Interdisciplinary studies, Geography, Risk factors

## Abstract

Public and school holidays have important impacts on population mobility and dynamics across multiple spatial and temporal scales, subsequently affecting the transmission dynamics of infectious diseases and many socioeconomic activities. However, worldwide data on public and school holidays for understanding their changes across regions and years have not been assembled into a single, open-source and multitemporal dataset. To address this gap, an open access archive of data on public and school holidays in 2010–2019 across the globe at daily, weekly, and monthly timescales was constructed. Airline passenger volumes across 90 countries from 2010 to 2018 were also assembled to illustrate the usage of the holiday data for understanding the changing spatiotemporal patterns of population movements.

## Abstract (summary)

**Table:** 

Measurement(s)	public holiday • school holiday • airline passenger volume
Technology Type(s)	digital curation
Factor Type(s)	geographic location • temporal interval
Sample Characteristic - Location	global

Machine-accessible metadata file describing the reported data: 10.6084/m9.figshare.17833922

## Background & Summary

Human populations are increasingly mobile, across both high- and low-income settings^1–3^. This also has substantial impacts on population distributions and dynamics, economies, social development and planning^4–9^. Domestic and international movements both show significant seasonal variations across countries^10,11^. This seasonality of human mobility has been attributed to multiple socioeconomic and climatic drivers across the globe^12^. Among them, some determinants play a greater role than others, including school terms, religious festivals, and national holidays^13,14^. For example, major national public or religious holidays are associated with shifts in the scope of travel and drive particularly strong fluctuations. Increasing volumes of travel are also commonly found around Christmas in Kenya, Namibia, and the United States, while travel decreases during Ramadan in Pakistan^13^. The ‘Golden week’ holidays of the National Day and Lunar New Year in China have also witnessed massive domestic and international movements^15^. Additionally, the seasonal changes of population densities between the major holiday period (July and August) and more traditional working periods (from September to June) in Portugal and France revealed clear spatial patterns: most cities are characterized by a large decrease in population densities during the holiday period, whereas less-populated areas and well-known tourist sites show large increases^16^.

The directionality of seasonal movements may also change over the course of a year, with the relative importance of particular routes changing. For example, travel from urban to rural areas increases in December in Namibia, while reverse population movements returning to cities occur in January, suggesting travel from urban areas for Christmas and back after the holiday^3,13^. Additionally, human mobility also changes seasonally with school terms and breaks. For instance, the largest increase in travel volume happens around Christmas in Kenya, Namibia and the United States, in line with school holidays^13^. Air traffic further tends to peak during long public holiday periods and school breaks over summer and winter that may cross months^17^, and holidays have also been revealed to coincide with seasonal mobility patterns measured by travel surveys, novel data sources (e.g., mobile phone call detail records), and social media, among others^13^. For example, anonymous cell phone data have been used to evaluate the change in traffic patterns caused by holidays, and patterns varied each day due to holiday effects (before the holiday, during the holiday, and after the holiday)^18^.

The homogenization and synchronicity of holidays across large regions of the globe could further facilitate pathogen spread through increased travel connectivity during national holidays and school breaks^13^. In particular, the mobility across countries during the coronavirus disease 2019 (COVID-19) pandemic has demonstrated how fast countries could be reached by an emerging pathogen and new variants^19–22^. For example, it was estimated that 5 million people including workers and students left Wuhan in China before the Lunar New Year holiday in January 2020^23^. Conversely, the timing of holidays and school breaks and travel restrictions may also reduce the close contact in some population groups, and then mitigate the spread of pathogens^24,25^.

Public and school holidays are therefore one of the main factors determining seasonal changes in human mobility and population dynamics, subsequently affecting the transmission of infectious diseases and many socioeconomic activities. However, the dates and timings of holidays may vary across years. Comprehensive and contemporary datasets of historical public and school holidays for nations around the world and their changes over time are critical for understanding the seasonality of human domestic and international movements. This has many potential applications across disciplines, from travel estimation, transport planning and management, resource allocation, to public health service provision and monitoring efforts. Despite this, the worldwide data of public and school holidays across years since 2010 have not been assembled into a single time series, free to obtain and easy to use.

This study aims to overcome this data gap identified by producing global, temporally explicit datasets of public and school holidays across countries and multiple years. Specifically, an open access archive of comprehensive datasets of public and school holidays across the world at the daily, weekly, and monthly level has been created. To illustrate the usage of holiday datasets for understanding seasonal patterns of human mobility, these time series of holidays are also compared against the statistics of airline passengers by month from 2010 to 2018, assembled in this study, and a dataset of monthly international airline ticket bookings across the world in 2015–2019, used in a previous COVID-19 research^22^. All products are available through the WorldPop website (https://www.worldpop.org/)^26–32^.

## Methods

**Fig. 1:**
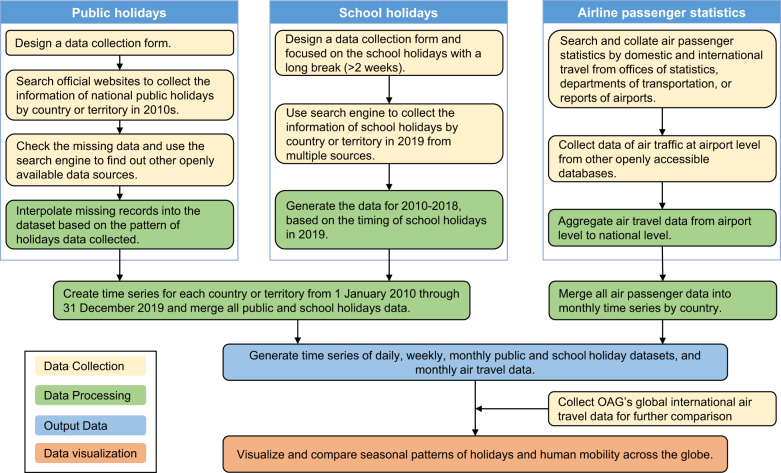
Schematic overview of the workflow to generate and analyse datasets. First, national public holidays from 2010 to 2019 were assembled, and school holidays in 2019 were collated to retrospectively generate school holidays from 2010 to 2018. Second, public and school holidays were merged to generate daily, weekly, and monthly time series. Third, the statistics and time series of monthly airline passenger numbers were collated to compared with the seasonal distribution of holidays across countries/territories/areas. Additionally, the Official Aviation Guide’s (OAG) global dataset of international air passenger ticket bookings from 2015 to 2019 were used for further assessing the correlations between seasonal mobility and holiday patterns.

Five steps were taken to assemble and validate the holiday datasets: 1) collating national public holidays for countries/territories/areas across the globe from 2010 to 2019; 2) collating school holidays in 2019 and retrospectively generating the school holiday data from 2010 to 2018; 3) merging and aggregating data of public and school holidays to generate time series at the daily, weekly, and monthly level; 4) collating monthly statistics of airline passengers travelling domestically and internationally, compared with the seasonal distribution of holidays; and 5) using the Official Aviation Guide’s (OAG) global dataset of international air passenger ticket bookings across 223 countries/territories/areas from 2015 to 2019, for further assessing the correlation between seasonal mobility and holiday patterns. Figure 1 provides a schematic overview of the study design.

### Public holiday data

Public holidays, also referred to as national holidays, bank holidays, or official holidays in different countries or regions, are usually non-working days of celebration or commemoration during the year established by law. Sovereign nations and territories generally observe public holidays based on events of significance to their history, such as the anniversary of a significant historical event (e.g., the National Day) or a religious celebration like Diwali, Christmas, Hanukkah, Ramadan, etc. Moreover, public holidays vary by country and sometimes by year. They can land on a specific day of the year or be tied to a certain week of the month, such as Thanksgiving, or follow other calendar systems like the Lunar Calendar. To commemorate special events, there has also been a number of ad hoc public holidays that were announced on short notice (<4 weeks), such as the 2-week-ahead announcement of extra holiday in the opening ceremony for the 2017 Southeast Asian Games held in Malaysia^33^.

In this study, we define national public holidays as ones established by law or announced by the corresponding authorities. A standardized data collection form was used to gather information on public holidays on a country-by-country basis from 2010 to 2019, with variables including: the name and ISO 3166 alpha-3 code of the country or territory, name and date of the holiday, and type of the holiday (e.g., public holiday, observance, special holiday, and half-day holiday). We also collected information on special working days occurring on weekends or non-working days that were officially and temporarily taken as replacements of non-working days during the week, such as the 7- or 8-day ‘Golden week’ holidays in China.

To assemble this dataset, we systematically searched information on public holidays for each country or territory via Google by using the search terms: [Public OR Federal OR official OR bank] AND [holidays] AND [Name of the country or territory] AND [Year]. Where data for a given area were available from multiple publicly available sources, we prioritized the data from official central or federal government/authority websites. If such data did not exist through official websites, other websites with openly available data were also considered, including: the Time and Date (www.timeanddate.com/holidays/), the Festivo (https://getfestivo.com/countries), the Office Holidays (https://www.officeholidays.com/countries), the Bhutanese Calendar (https://www.bhutanesecalendar.com), and the Nager.Date (https://date.nager.at/). However, comparing with the data in the latter half of the 2010s, data spanning 2010–2014 were not widely available. We therefore identified missing data in the dataset by comparing the number of holidays by year for each region. For missing data on public holidays in a year that were tied to a specific day of each year, we interpolated the records into the dataset. For public holidays with variable dates across the years, we inferred dates where possible if they landed on a certain day of the week in a certain month or followed other calendar systems like the Lunar Calendar. Finally, we merged these interpolated and inferred public holiday within the final dataset^27^.

### School holiday data

School holidays (also referred to as vacations, breaks, and recesses) are the periods during which primary and secondary schools are closed or no classes or other mandatory activities are held. The dates and periods of school holidays vary considerably throughout the world, and there is usually some variation even within the same jurisdiction, with governments sometimes legislating only the total number of school days required. In this study, we defined school holidays for primary and secondary schools only, excluding higher education, such as universities. Because short holidays or mid-term breaks commonly overlap with public holidays (e.g., the Easter or Thanksgiving), we focused on long school holidays with breaks lasting more than 2 weeks, e.g., summer and winter holidays between academic years. We similarly created a standardized form to collect and collate data, with the variables including name and ISO 3166-alpha3 code of country or territory, name of the school holiday (e.g., spring/summer/autumn/winter holiday, or break between school years), the first date and the last date of the holiday.

We systematically searched the information on school holidays for each single country or territory in Google by using the search terms: [school] AND [Holiday OR Break OR Term] AND [Name of the country or territory] AND [Year]. We prioritized data from official central or federal government/authority websites. If these data were unavailable at the country level, we collected information on school holidays for capital regions, announced by local governments or educational departments. For example, school holidays in China varied across provinces, so we therefore relied on information about school holidays within Beijing. For those countries without available data from official websites, we also searched publicly available data from websites including: the School Holidays (https://school-holidays.net/), the Public Holidays Asia (https://publicholidays.asia/), the School Holidays Europe (https://www.schoolholidayseurope.eu/), and the Holiday Calendar (https://holidaycalendar.com/).

Due to variability in dates of school academic years and terms across schools, regions and countries, median dates were used for discrepancies in beginning and end dates of school holidays across regions within a country for the same year. However, historical data on school holidays are not widely available from websites, and school breaks in each country vary by year, but generally occur during the same season. For example, Namibia and Kenya have school breaks in April, August, and December/January, and Pakistan has a single long break from July–August^13^. Therefore, we firstly collated information on school holidays in 2019, and then estimated beginning and end dates of school holidays between 2009 and 2018 using the same information from 2019. Of note, if the beginning dates in 2010–2018 were on Thursday or Friday, they were adjusted to the nearest Monday, and if the end dates in 2010–2018 were on Monday or Tuesday, they were adjusted to the nearest Sunday^28^.

### Holiday time series

We then created time series on a daily basis for each country or territory from 1 January 2010 through 31 December 2019, and generated the fields of year, month, and week number in each year. The time series were merged with the public holiday data to decide whether each day of the year was a holiday or not. Similarly, this was merged with the school holidays data to identify whether the day was a school holiday or not. We added a variable (i.e., hl_sch) to indicate whether each day included a public or school holiday^29^. Finally, the daily time series were aggregated to generate weekly time series^30^ and monthly time series^31^, by calculating how many days in each week or month contained school or public holidays.

### Airline passenger statistics

To understand the impact of holidays on seasonal mobility and illustrate the usage of holiday datasets, we also collated monthly statistics of airline passengers travelling domestically and internationally, as censuses and surveys commonly do not collect the data of seasonal population movements across countries. The air travel data span 2010 to 2018 were systematically searched and collected from the National Offices of Statistics or Departments of Transportation across continents and countries^34–40^. We also used publicly accessible databases of airline passengers at the airport level from the Anna Aero (https://www.anna.aero/databases/). All data were then aggregated from the airport level to national level. We merged data into a time series at the monthly level^32^, which included the following variables: ISO 3166-alpha3 code of each country or territory, year, month, total number of air passengers (obtained from statistics), number of internal air travellers, number of international air travellers, and the total number of airline passengers using data obtained from other sources such as Anna Aero.

As the statistics of airline passengers might not be available for all countries across the world, particularly in the low- and middle-income settings, we further used OAG’s global dataset (https://www.oag.com/) of international travellers based on air ticket bookings from 2015 to 2019, for investigating the correlations between holidays and mobility for countries that were not covered by air traffic statistics assembled by this study. The OAG data of international traffic flows have been used in our previous study to understand the international spreading risk of COVID-19 at the early stage of pandemic from December 2019 to May 2020^22^. The data obtained from OAG are not publicly available due to stringent data licensing agreements, but the information on the process of requesting access to the dataset that supports the findings of this study is available from the corresponding author.

## Data Records

The datasets of public and school holidays and airline passenger statistics assembled by this study are publicly and freely available through the WorldPop Repository (https://www.worldpop.org/)^27–32^. A collection of these datasets with DOIs has been compiled and described in Table 1.

**Table 1:** Name and variables of time series datasets for global holidays and air travel.

Name	Variables and explanations	File Format	University of Southampton DOI
Global Public Holidays Data, 2010–2019^27^	ADM_name (Name of the country or territory), ISO3 (ISO 3166 alpha-3 code of the country or territory), Date (DD/MM/YYYY), Name (name of public holiday), and Type (type of public holiday).	csv	10.5258/SOTON/WP00689
Global School Holidays Data, 2010–2019^28^	ADM_name, ISO3, Name_short (name of school holiday), Date_b19 & Date_e19 (the beginning date and end date of a school holiday in 2019), …, Date_e10 & Date_e10 (the beginning date and end date of a school holiday in 2010).	csv	10.5258/SOTON/WP00690
Daily Time Series of Global Public and School Holidays, 2010–2019^29^	ISO3, Date, ADM_name, Year (2010–2019), Month (1–12), Week (week numbers in a year), Day (day numbers in a month: 1–31), holiday (public holiday: 1 - Yes, 0 - No), school (school holiday: 1 - Yes, 0 - No), and hl_sch (public or school holiday: 1 - Yes, 0 - No).	csv	10.5258/SOTON/WP00691
Weekly Time Series of Global Public and School Holidays, 2010–2019^30^	ISO3, ADM_name, Year, Week, holiday, and hl_sch.	csv	10.5258/SOTON/WP00692
Monthly Time Series of Global Public and School Holidays, 2010–2019^31^	ISO3, ADM_name, Year, Month, holiday, and hl_sch.	csv	10.5258/SOTON/WP00693
Monthly Volume of Airline Passengers in 90 countries, 2010–2018^32^	ISO3, Year, Month, Total (total number of air passengers in thousands, obtained from official statistics; NA – data non-available), Domestic (number of internal air passengers in thousands for a country, obtained from official statistics), International (number of international air passengers in thousands, obtained from official statistics), and Total_OS (total number of air passengers in thousands, obtained from other openly available data sources).	csv	10.5258/SOTON/WP00694

## Technical Validation

All data collected, assembled and used were (i) already validated by the corresponding data collector, owner and/or distributor, (ii) visualised to present their spatiotemporal and spatial patterns, and (iii) further quality-checked, in the framework of this project, for the synchronicity and correlations between holiday patterns and seasonal human mobility derived from air travel datasets.

### Public and school holidays

**Fig. 2:**
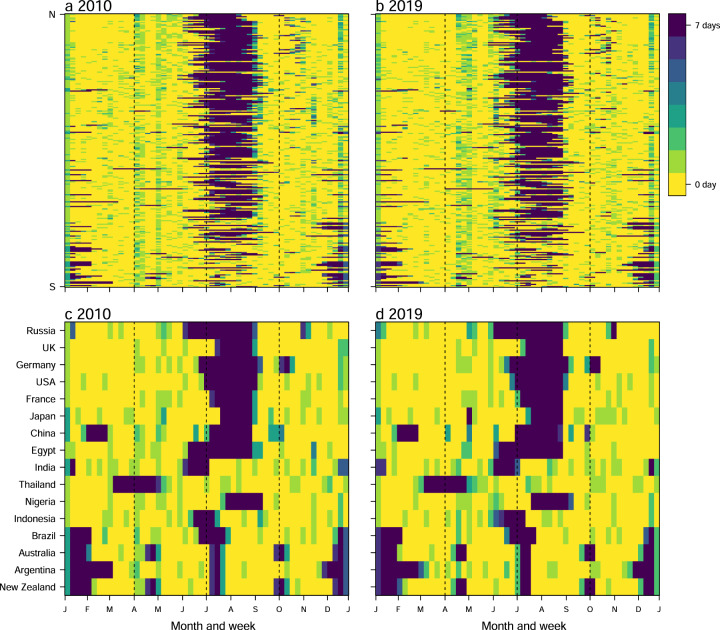
Heatmaps of weekly time series of holidays in 2010 and 2019. Each row in the heatmap represents a country/territory, sorted by the latitudes of their capitals from North to South. (**a**) and (**b**) present the number of holidays by week in 2010 and 2019 across the world, respectively. (**c**) and (**d**) show weekly holiday patterns for 16 countries in 2010 and 2019, respectively.

**Fig. 3:**
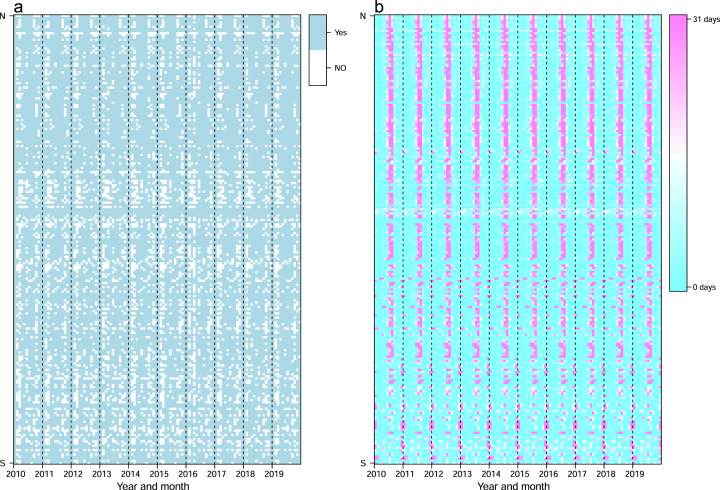
Heatmaps of monthly time series of holidays across the world in the 2010–2019 period. Each row in the heatmap represents a country/territory/area, sorted by the latitudes of their capitals from North to South. (**a**) The months with or without public and school holidays. (**b**) The days of holidays for each month.

**Fig. 4:**
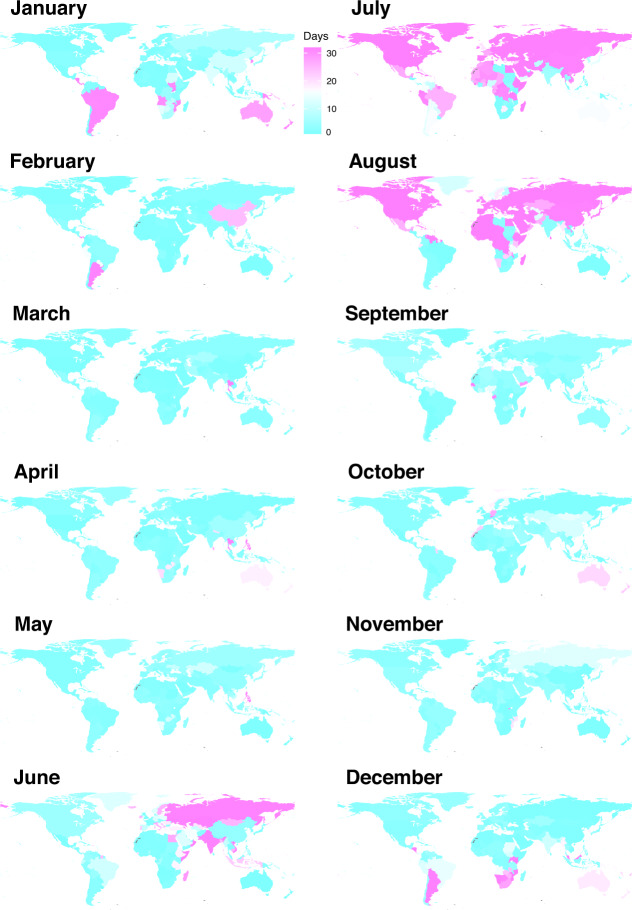
Average number of public and school holidays by month and country/territory/area in the 2010–2019 period. The regions without data are filled with grey colour.

Time series data of public and school holidays were assembled for 232 countries/territories/areas across the world, with noticeable seasonality from 2010 to 2019 (Figs. 2 and 3) and across regions (Fig. 4). We checked the number and seasonal patterns of holidays over years by country, as holidays generally occur at similar periods across years in each country. Our datasets present clear seasonality in holidays by country, but the timings of some holidays based on the Lunar Calendar, for example, change by year. However, some countries further move non-working days from the weekend to the weekday and combine with public holidays to allow for longer holidays. For instance, China has a ‘golden week’ with 7 to 8 days of national holiday, including the Chinese New Year in January/February, and the Mid-Autumn Festival and National Day in late September and early October, facilitating long-distance family visits (Fig. 2).

Winter and summer school holidays contributed markedly to holiday seasonality. Most countries have summer holidays or vacations as the longest break in the school year, lasting between 5 and 14 weeks. For example, summer holidays in Ireland, Italy, Lithuania and Russia normally last three months, compared to 6-8 weeks in the United Kingdom, and the Netherlands and Germany from June to August. The summer break in the southern hemisphere commonly lasts 6–8 weeks from December to February, overlapping with Christmas and New Year’s Day holidays, while the winter break in predominantly Christian countries in the northern hemisphere normally last for about 1–3 weeks surrounding Christmas (Fig. 2). Additionally, in countries with a history of Christianity, the Easter holiday is a school break that takes place in the northern hemisphere’s spring and in the southern hemisphere’s autumn, with the date varying by country and level of schooling (e.g., primary versus secondary). For South-East Asian countries celebrating the Spring Festival or Lunar New Year, there is also a long school break towards the beginning of the year, lasting between 4 and 6 weeks around January and February.

### Holidays and seasonal population mobility

**Fig. 5:**
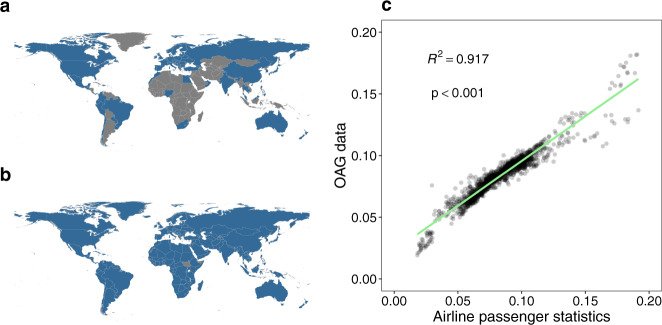
The availability of seasonal air travel data. (**a**) Airline passenger statistics in 90 countries/territories/areas, collated by this study from openly available data sources. (**b**) The availability of international air travel data based on passenger bookings, obtained from the Official Aviation Guide (OAG). The regions without data in maps are filled with grey colour. (**c**) The correlation between the statistics of international airline passengers assembled in this study and OAG data. The air travel data are presented as the proportion of international travellers among each year and country/territory/area. The green solid line represents linear regression fit, with p and *R*^2^ values provided.

**Fig. 6:**
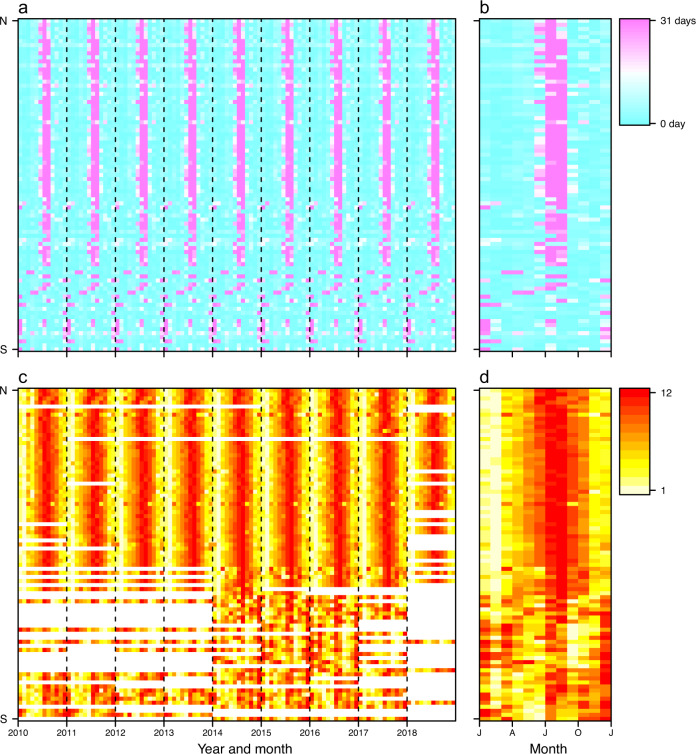
Seasonal patterns of holidays and air travel for regions with available airline passenger statistics in 2010–2018, assembled by this study. (**a**) Days of holidays in each month. (**b**) The seasonality of holidays, presented by the average number of days of public and school holidays in the same period across years. (**c**) The rank of monthly volume of domestic and international airline passengers. Months with higher volumes have a higher rank (from the lowest to the highest: 1–12) in each year. Months without data are coloured white. (**d**) The seasonality of air travel, presented by average rank of airline passenger numbers for the same period across years. Each row in the heatmap represents a country/territory/area, sorted by the latitudes of their capitals from North to South.

**Fig. 7:**
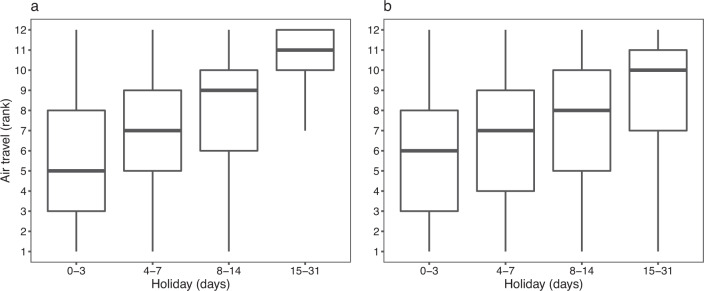
Boxplots of the monthly volume of airline passengers by the duration of holidays across the world. (**a**) Airline passenger statistics in 90 countries/territories/areas, collated by this study from openly available data sources. (**b**) International air travel data based on passenger bookings, obtained from the Official Aviation Guide. The monthly volume of air travel was transformed as the rank. Months with higher volumes of airline passengers have a higher rank (from the lowest to the highest: 1–12) in each year and country.

**Fig. 8:**
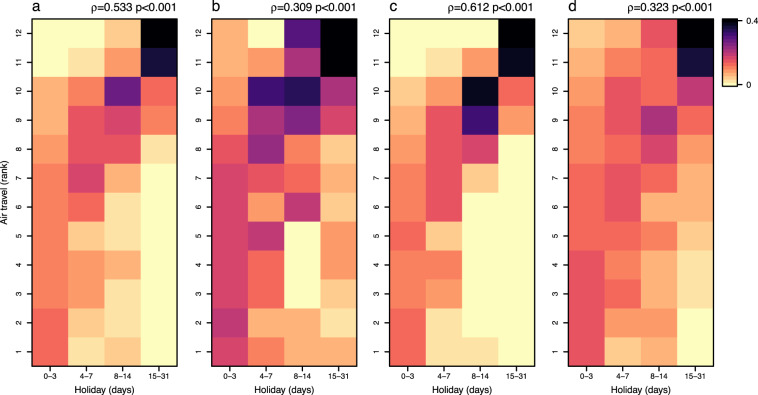
Correlations between the duration of holidays and the volume of air travel by month, 2010–2018. (**a**) Domestic and international travel, (**b**) domestic travel, and (**c**) international travel, based on the air travel statistics collated by this study from openly available data sources. (**d**) International air travel data based on passenger bookings, obtained from the Official Aviation Guide. The monthly volume of air travel was transformed as the rank. Months with higher volumes of travellers have a higher rank (from the lowest to the highest: 1–12) in each year and country/territory/area. The colour of each tile means the proportion of months in each air travel rank over the total number of months in each category of the duration of holidays across the world. The Spearman’s rank correlation coefficients (ρ) and p values are provided to assess the relationship between the duration of holidays and the volume of air travel.

We collated the statistics of airline passengers for a total of 90 countries/territories/areas from publicly available data sources from 2010 to 2018 (Figs. 5a and 6), with the majority of countries in Europe, North America, and East Asia. Comparing air travel data obtained from official statistics versus other sources, we found slight discrepancies. This might be due to a number of factors, including: i) some countries, e.g., Australia and Canada, only reporting monthly statistics of traffic for major airports or airlines; or ii) duplication of air passengers due to data collection from a variety of data sources. For instance, those airport-level data including total number of incoming and outgoing passengers had being aggregated from airport level to national level, and domestic passengers being at more than one airport in the same country might be counted twice, especially in geographically vast countries, e.g., USA, Canada, or China. To overcome these issues, we only used data from other sources at the airport level for countries and years without official statistical data available, and then transformed the actual monthly traffic data to relative values by ranking monthly volumes of airline passengers within each year and country. We found that more people travelled around July – August in the northern hemisphere, while a high volume of air travel occurred in July – August and December – January in the southern hemisphere (Fig. 6). These seasonal patterns demonstrated high correlations between human mobility and the duration of public and school holidays, for both domestic and international travel (Figs. 7 and 8).

**Fig. 9:**
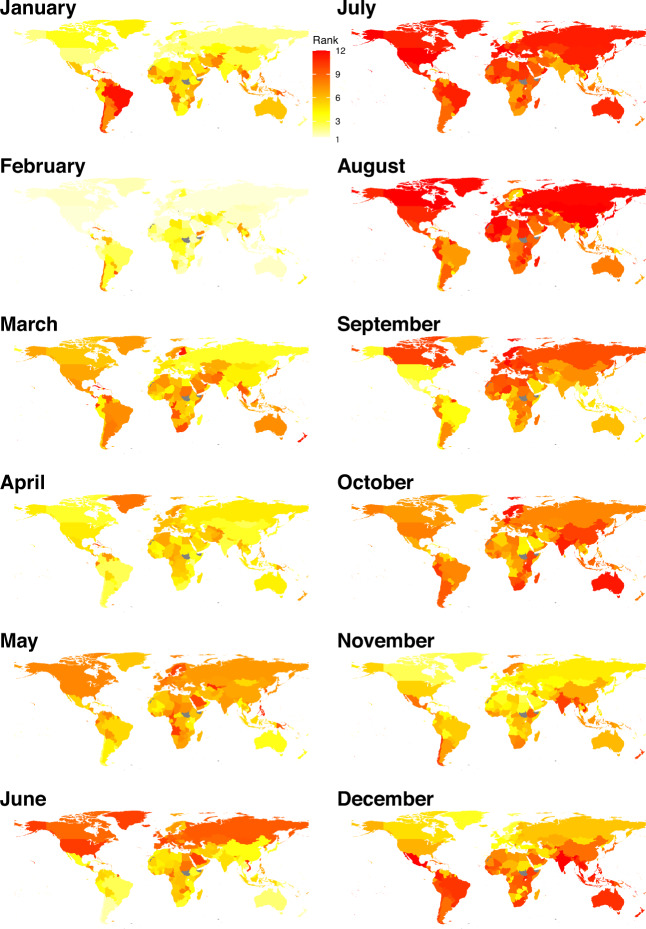
Average rank of international air travel by month and country/territory/area in the 2015–2019 period. International air travel data were based on passenger bookings, obtained from the Official Aviation Guide. The regions without data are filled with grey colour.

**Fig. 10:**
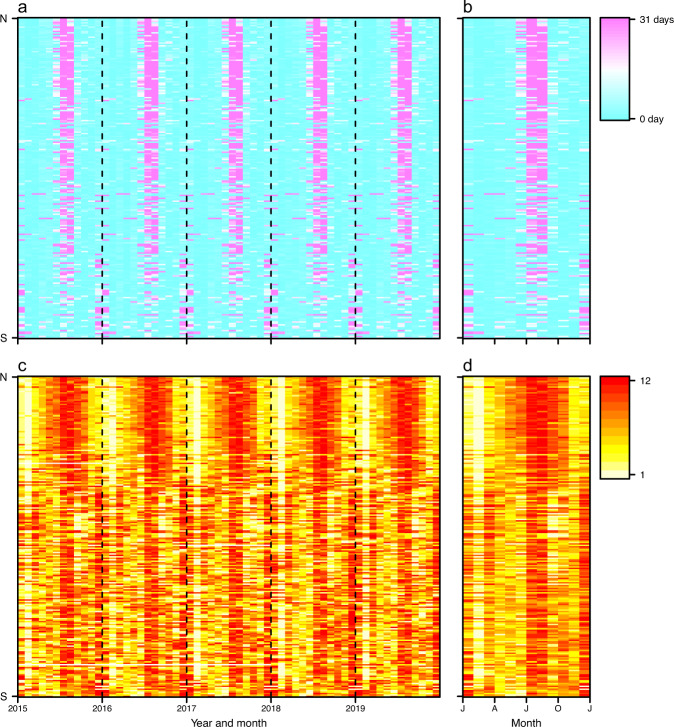
Seasonal patterns of holidays and international travel for air passengers across 223 countries/territories/areas from 2015 to 2019. (**a**) Days of holidays in each month. (**b**) The seasonality of holidays, presented by the average number of days of public and school holidays in the same period across years. (**c**) The rank of monthly volume of international air travellers, obtained from the Official Aviation Guide. Months with higher volumes have a higher rank (from the lowest to the highest: 1–12) in each year. Months without data are coloured white. (**d**) The seasonality of air travel, presented by average rank of traveller counts for the same period across years. Each row in the heatmap represents a country/territory/area, sorted by the latitudes of their capitals from North to South.

However, only limited data of air travel statistics across multiple years were available for countries in Africa, South America, West and Southeast Asia (Fig. 5a). To overcome this limitation, an extra dataset of global international air travel covering almost all countries from 2015 to 2019 were obtained from the OAG (Fig. 5b). We found that this dataset highly correlated with the statistics of international airline passengers assembled in this study (Fig. 5c). The OAG dataset also showed a clear seasonal pattern and there were more people travelling during the period of longer holidays, i.e., July – August across the world and December – January in the southern hemisphere (Figs. 9 and 10). A significant Spearman’s rank correlation coefficient was also found between international travel and holidays across the world (Figs. 7b and 8d).

## Usage Notes

The archive provides ready to use time series at daily, weekly and monthly temporal resolutions and at national spatial scales. This compilation of datasets can facilitate a variety of uses across settings, from quantifying and predicting seasonal population movements, to modelling disease transmission dynamics and interventions, as well as air traffic predictions and estimation of their socioeconomic impact. For example, using the holiday datasets assembled in this study, a recent research has explored how a set of broadly available covariates can describe the seasonal dynamics of population movements in Kenya, therefore enabling better modelling of seasonal mobility across low- and middle-income settings^41^. They found that Kenyan mobility peaked in August and December, closely corresponding to school holiday seasons, and the holiday was found to be an important predictor in the model. Additionally, we can quantify the contribution of holidays on seasonal population mobility derived from traditional or new data sources, e.g., mobile phone call detail records and internet check-in location history data, and statistical and mathematical models using holiday data can be built to predict future mobility across space and over time. Moreover, understanding and predicting human movements using these data should ensure other relevant covariates are used, e.g., temperature and tourism activities. For instance, summer is the most popular season for mobility in most countries in Europe due to two factors: i) the summer months, and particularly August, are those when most people or families traditionally go on holidays, when many activities are closed (e.g., education) or have reduced activity (e.g., manufacturing); ii) the warm temperatures are a very important pull factor for holidays in the majority of these regions. Nonetheless, there are some exceptions. The winter season is popular in some alpine regions due to favourable natural conditions for winter sports/activities, such as skiing. Lastly, domestic and international travel restrictions and social distancing policies aimed at containing outbreaks will likely significantly alter mobility patterns, regardless of climate and holiday factors, and should therefore be accounted for in any models using these data^25,42^.

Of note, week numbers in the weekly time series datasets were calculated by year and contain a week 0 for some years; the days of that week should therefore be included in the last week of the preceding year. Further, some countries combine public holidays with weekends to create 3-day or longer holidays, and these holidays may have a stronger impact on mobility than single-day holidays. Lastly, working days or the weekend are not identical across the globe. For example, Nepal has a six-day working week from Sunday to Friday, and the weekend in many Middle East and North Africa countries occur on Friday and Saturday. These country level nuances and timings of holidays with weekends should be accounted for where possible when performing single country analyses.

These analyses and data are subject to some limitations. Firstly, not all data are accessible from official websites or other publicly available sources, especially the holidays in the first half of the 2010–2020 period. We therefore interpolated data for these time periods based on reoccurring holidays across years, where possible. However, it is possible we did not accurately reflect those changes due to the replacement of holidays on the weekend by moving them from weekend to workday. Secondly, we calculated median dates for the beginning and end date of school holidays nationally, which might not represent the actual duration of school breaks at subnational or local levels. Additionally, many schools have the flexibility to adjust their school terms dates, e.g., adding inset days or as a result of snow days, but our datasets will likely not reflect these changes, as they occur organically through individual schools, events or jurisdictions. Thirdly, only air traffic data were collated to understand the seasonality of human movements and potential applications of the public and school holiday datasets. Other traditional data sources such as travel surveys, combining with data from novel sources, e.g., mobile phone data, social media, and internet check-in data, might be valuable in more accurately capturing human mobility across various temporal and spatial scales. In future research, these data sources can be used to better examine driving factors of human mobility, including identification of public and school holidays, public health interventions, natural disasters, and climate changes, among others.

## Data Availability

R version 3.6.1 (R Foundation for Statistical Computing, Vienna, Austria) was used to manage data and perform analyses in this study. The code used to generate datasets and plots is available for download from the repository on GitHub at https://github.com/LaiShengjie/Holiday.
